# Protection of Primary Dopaminergic Midbrain Neurons by GPR139 Agonists Supports Different Mechanisms of MPP^+^ and Rotenone Toxicity

**DOI:** 10.3389/fncel.2016.00164

**Published:** 2016-06-28

**Authors:** Kirsten Bayer Andersen, Jens Leander Johansen, Morten Hentzer, Garrick Paul Smith, Gunnar P. H. Dietz

**Affiliations:** ^1^Department of Neurodegeneration, H. Lundbeck A/SValby, Denmark; ^2^Department of Molecular Screening, H. Lundbeck A/SValby, Denmark; ^3^Department of Discovery Chemistry 2, H. Lundbeck A/SValby, Denmark

**Keywords:** Parkinson’s disease model, G protein-coupled receptor, neurodegeneration, drug screening, neuroprotection, cell-based assay, neurotoxin, apoptosis

## Abstract

The G-protein coupled receptor 139 (GPR139) is expressed specifically in the brain in areas of relevance for motor control. GPR139 function and signal transduction pathways are elusive, and results in the literature are even contradictory. Here, we examined the potential neuroprotective effect of GPR139 agonism in primary culture models of dopaminergic (DA) neuronal degeneration. We find that *in vitro* GPR139 agonists protected primary mesencephalic DA neurons against 1-methyl-4-phenylpyridinium (MPP^+^)-mediated degeneration. Protection was concentration-dependent and could be blocked by a GPR139 antagonist. However, the protection of DA neurons was not found against rotenone or 6-hydroxydopamine (6-OHDA) mediated degeneration. Our results support differential mechanisms of toxicity for those substances commonly used in Parkinson’s disease (PD) models and potential for GPR139 agonists in neuroprotection.

## Introduction

The G protein-coupled receptor (GPCR) superfamily is the largest group of cell surface receptors. An estimated 36% of drugs approved by the United States Food and Drug Administration during the last three decades target GPCRs, making them the most common drug target (Rask-Andersen et al., 2011). Many GPCRs have been cloned without knowledge of their function and ligands (Davenport et al., 2013) and are likely to cover a number of future drug targets. One of these is the G protein coupled-receptor 139 (GPR139). It was found to belong to the class A GPCRs and to be highly conserved through different species including human, mouse, rat, chicken, fugu and zebrafish (Gloriam et al., 2005). Recently, we have identified L-Trp and L-Phe as the first potential endogenous agonists of GPR139 (Isberg et al., 2014) which was subsequently confirmed by researchers at Jannsen (Liu et al., 2015). In humans and mice, *Gpr139* is expressed specifically in distinct areas of the CNS, including the lateral aspect of the striatum (Matsuo et al., 2005; Süsens et al., 2006). Moreover, preliminary analysis suggested that *Gpr139* knockout mice display deficits in locomotion, balance and sensorimotor tasks (Murphy and Croll-Kalish, 2004). The expression pattern of *GPR139* and the initial phenotypic analysis of the loss-of-function are consistent with a role in the modification of locomotor activity. Recently, while this study was in progress, it was found that GPR139 agonists reduce locomotor activity in rats (Liu et al., 2015). Variations in the *Gpr139* locus have been linked to inattention (Ebejer et al., 2013) in ADHD patients and to schizophrenia (Castellani et al., 2014).

Parkinson’s disease (PD) is the second-most prevalent neurodegenerative disease (de Lau and Breteler, 2006). The etiology of PD is still unknown in most cases, but the characteristic motor symptoms of PD are primarily due to the loss of neurons of the nigrostriatal dopaminergic (DA) pathway. Treatment of PD at present is symptomatic, induces side effects and does not stop disease progression. There is thus a huge need for innovative and new treatment strategies (Aquino and Fox, 2015; Bastide et al., 2015; Ossig and Reichmann, 2015).

The neurotoxin 1-methyl-4-phenyl-1,2,3,6-tetra-hydropyri-dine (MPTP) is converted into the active 1-methyl-4-phenyl-pyridinium ion (MPP^+^) in the brain (Przedborski and Vila, 2003) and can produce similar biochemical and neuropathological defects as observed in PD patients (Langston et al., 1984). These include the progressive loss of DA neurons in the substantia nigra pars compacta (SNpc) and the decrease of striatal dopamine levels. Therefore, it is one of the most widely used experimental models for sporadic PD (Przedborski and Vila, 2003). Lipophilic MPTP passes the blood-brain barrier (BBB) and cellular membranes. In astrocytes, monoamine oxidase B converts MPTP into MPP^+^ (Ransom et al., 1987). MPP^+^ is taken up into DA neurons by the dopamine transporter (DAT; Javitch et al., 1985; Mayer et al., 1986) inhibiting mitochondrial complex I (Tipton and Singer, 1993). It promotes ATP depletion and generation of reactive oxygen species (ROS; Rossetti et al., 1988), which can activate apoptotic pathways (Przedborski et al., 2004). Rotenone is another mitochondrial complex 1 inhibitor applied in PD models (Betarbet et al., 2000), but unlike MPP^+^ it is lipophilic and can therefore readily cross the cell membranes. 6-Hydroxydopamine (6-OHDA) is taken up both by the DAT and the noradrenergic transporter, and therefore induces cell death in both DA and noradrenergic neurons (Luthman et al., 1989). Like MPP^+^ and rotenone 6-OHDA is used for both *in vitro* and *in vivo* models for investigations of the underlying mechanism of PD (Ungerstedt, 1968; Sachs and Jonsson, 1975; Blesa and Przedborski, 2014).

Recently, three agonists and an antagonist were developed as tools to further examine GPR139 function, one of which has been described (Shi et al., 2011). Here, we examined whether GPR139 signaling could modify toxicity of those most commonly used toxins used in PD models. We assessed toxin resistance of primary DA cells pre-treated with the GPR139 agonists, and whether protection by GPR139 signaling could be blocked by co-incubation with the antagonist.

## Materials and Methods

### Compounds

#### Compound 1

The GPR139 agonist compound 1, 2-(3, 5-Dimethoxybenzoyl)-N-(1-naphthyl)-hydrazinecarboxamide, with an EC_50_ of 39 nM has been described earlier (Shi et al., 2011). EC_50_ and IC_50_ for all compounds were determined as described (Shi et al., 2011).

#### NMR and MS for the Preparation of Compound 2 and 3

The ^1^H NMR spectra were recorded on a Bruker Avance AV (500 MHz) with tetramethylsilane (TMS) as internal standard. ESI-MS spectra were measured with a Thermo Finnigan LCQ14ECAXP or a PE-Sciex API 1SO-Ex. Low-resolution EI-MS was measured on a MAT-95 spectrometer and high resolution ESI-MS measured with a MAT-77 spectrometer or using a Bruker micro-TOF. NMR spectra were obtained using d_6_-DMSO or CDCl_3_ as solvent. Chemical shifts are expressed as δ units (ppm) relative to TMS as internal standard. The abbreviations s, d, t, m and br refer to singlet, doublet, triplet, multiplet and broad signal.

#### Preparation of (2-Naphthalen-1-yl-acetylamino)-Acetic Acid Ethyl Ester (Figure 1)

1-Naphthaleneacetic acid (3.03 g, 16.3 mmol) was dissolved in dichloromethane (83.5 mL). Glycine ethyl ester hydrochloride (2.5 g, 18 mmol) and triethyamine (4.76 mL, 34.2 mmol) were added and the solution cooled to 0°C under an argon atmosphere. N-(3-Dimethylaminopropyl)-N′-ethylcarbodiimide hydrochloride (3.43 g, 17.9 mmol) was added and the reaction was stirred for 2 h at 0°C and then at room temperature for 16 h. The reaction was washed with saturated NaHCO_3_ (1 × 100 mL), 1M HCl (1 × 100 mL) and then brine (100 mL). The separated organic layer was dried (MgSO_4_), filtered and concentrated in vacuo to give the desired product. Yield: 3.6 g, 81%.

**Figure 1:**
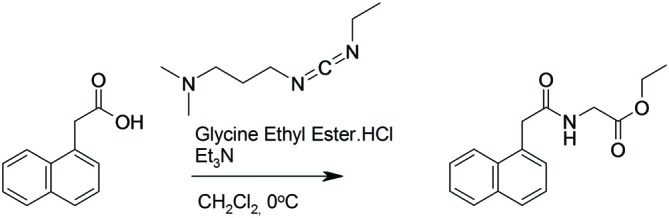
**Synthesis of (2-naphthalen-1-yl-acetylamino)-acetic acid ethyl ester.** 1-Naphthaleneacetic acid was dissolved in dichloromethane. Glycine ethyl ester hydrochloride and triethyamine were added and the solution cooled to 0°C under an argon atmosphere. N-(3-Dimethylaminopropyl)-N′-ethylcarbodiimide hydrochloride was added and the reaction was stirred for 2 h at 0°C and then at room temperature for 16 h.

^1^H NMR (500 MHz, CDCl_3_) δ 8.0 (d, 1H), 7.91 (d, 1H), 7.84 (d, 1H), 7.59–7.50 (m, 2H), 7.50–7.43 (m, 2H), 5.53 (s, 1H), 4.14–4.07 (m, 4H), 3.93 (d, 2H), 1.19 (t, 3H).

#### Preparation of (2-Naphthalen-1-yl-acetylamino)- Acetic Acid (Figure 2)

(2-Naphthalen-1-yl-acetylamino)-acetic acid ethyl ester (4.0 g, 10 mmol) was dissolved in ethanol (100 mL). Sodium hydroxide (2 M, 22.1 mL) was added and the reaction was stirred for 16 h at room temperature. The reaction mixture was concentrated in vacuo and 2M HCl added until the mixture was acidic. The product precipitated and was filtered. The solid was washed with water and ether and dried in a vaccuum. Yield: 2.85 g, 80%.

**Figure 2:**
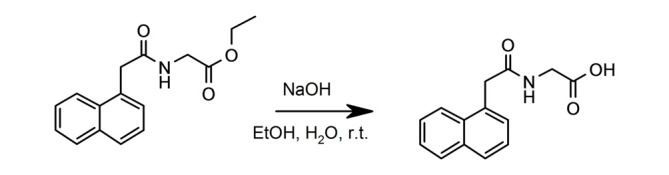
**Synthesis of (2-naphthalen-1-yl-acetylamino)-acetic acid.** (2-Naphthalen-1-yl-acetylamino)-acetic acid ethyl ester was dissolved in ethanol. Sodium hydroxide was added and the reaction was stirred for 16 h at room temperature.

^1^H NMR (500 MHz, DMSO-d_6_) δ 8.51 (d, 1H), 8.09 (d, 1H), 7.91 (d, 1H), 7.80 (br s, 1H), 7.51)br s, 2H), 7.44 (br s, 1H), 3.10 (s, 2H), 3.78 (s, 2H).

#### Preparation of Compound 2: N-[2-(3-Dimethylamino-pyrrolidin-1-yl)-2-oxo-ethyl]-2-Naphthalen-1-yl-acetamide (Figure 3)

(2-Naphthalen-1-yl-acetylamino) acetic acid (600 mg, 2.46 mmol) was dissolved in dichloromethane (75 mL) and N,N-diisopropylethylamine (1.1 mL, 6.2 mmol) under an nitrogen atmosphere at room temperature then and cooled to 0°C. To the solution was added (3-dimethylamino)pyrrolidine (235 mg, 2.06 mmol) and N,N′-dicyclohexylcarbodiimide (848 mg, 4.1 mmol). The mixture was stirred overnight at room temperature. The reaction mixture was washed twice with aq. NaOH (1M), brine (50 mL), dried with MgSO_4_, filtered and concentrated in vacuo. The crude product was purified by flash chromatography using heptane/ethyl acetate/trimethylamine/methanol (1:1:0.05:0.1). The purified product was isolated as a solid. Yield: 520 mg, 62%.

**Figure 3:**
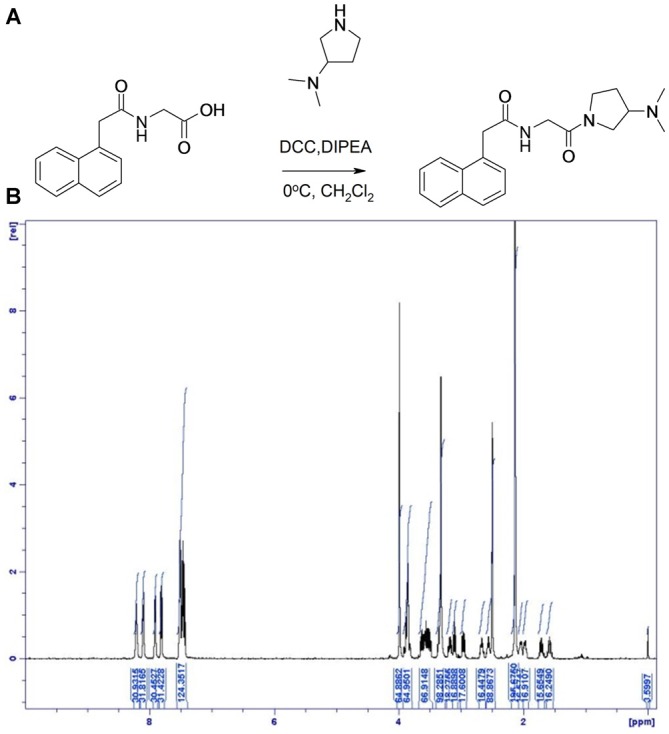
**Synthesis of Compound 2. (A)** ((1-Naphthylacetyl)Amino)acetic acid was dissolved in dichloromethane and N, N-diisopropylethylamine at room temperature and cooled to 0°C. To the solution was added (3-dimethylamino)pyrrolidine and N, N’-dicyclohexylcarbodiimide. **(B)** ^1^H NMR spectrum of compound 2, with specifications provided in “Materials and Methods” Section.

LC-MS [M + H]^+^ found at 340.3. ^1^H NMR (500 MHz, DMSO- d_6_) δ 8.24 (1H, dd), 8.12 (1H, dd), 7.93 (m, 1H), 7.83 (d, 1H), 7.56–7.43 (m, 3H), 4.00 (s, 2H), 3.88 (m, 2H), 3.65–3.5 (m, 2H), 3.36 (s, 4H), 3.18 (m, 0.5H), 3.11 (m, 0.5H), 3.01–2.95 (m, 2H), 2.67 (dd, 0.5H), 2.56 (dd, 0.5H), 2.14 (d, 1H), 2.05 (m, 0.5H), 1.98, (m, 0.5H), 1.72 (m, 0.5H), 1.57 (m, 0.5H), 0.98 (m, 1H).

The GPR139 surrogate agonist compound 2 had an EC_50_ of 530 nM.

#### Preparation of Compound 3: N-[(2-Methoxy-ethyl)-Methyl-carbamoyl]-methyl-2-Naphthalen-1-yl-acetamide (Figure 4)

(2-Naphthalen-1-yl-acetylamino) acetic acid (400 mg, 2 mmol), N-(2-Methoxyethyl)methylamine (138 mg, 1.55 mmol) and triethylamine (0.433 mL, 3.11 mmol) were dissolved in dichloromethane (10 mL) and cooled to 0°C. N-(3-Dimethylaminopropyl)-N′-ethylcarbodiimide hydrochloride (447 mg, 2.33 mmol) was added and the reaction was stirred at 0°C for 2 h and then for 16 h at room temperature. The reaction was washed with saturated sodium bicarbonate, 1M HCl and brine. The organic layer was dried with MgSO_4_ and the compound was concentrated in vacuo. The crude product was purified by flash chromatography eluting with heptane/ethyl acetate 1:1. Yield: 0.33 g, 68%.

**Figure 4:**
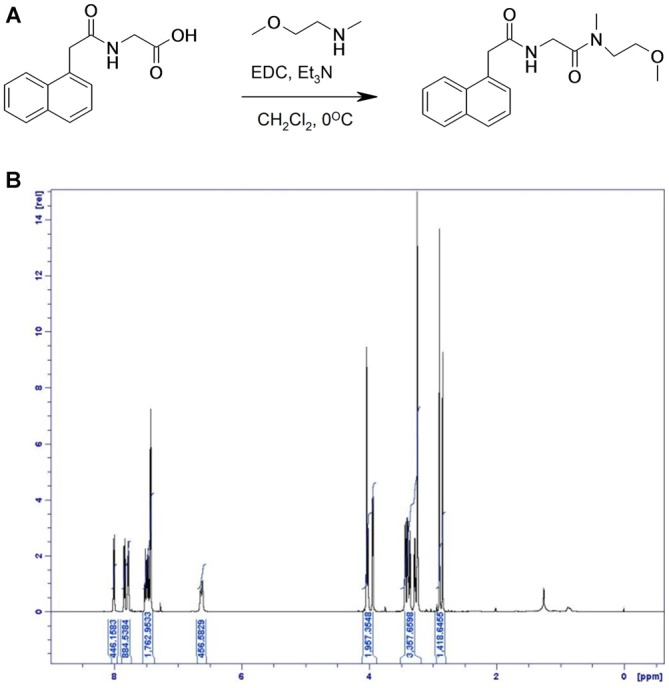
**Synthesis of compound 3. (A)** [(1-Naphthylacetyl)amino]acetic acid, N-(2-Methoxyethyl)methylamine and triethylamine were dissolved in dichloromethane and cooled to 0°C. N-(3-Dimethylaminopropyl)-N′-ethyl-carbodiimide hydrochloride was added and the reaction was stirred at 0°C and then at room temperature. **(B)** ^1^H NMR spectrum of compound 3, with specifications provided in “Materials and Methods” Section.

LC-MS (M + H)^+^ found at 315.3. ^1^H NMR (500 MHz, CDCl_3_) δ 8.00 (d, 1H), 7.83 (d, 1H), 7.78 (m, 1H), 7.50–7.44 (m, 2H), 7.43 (d, 2H), 6.61 (d, 1H), 4.05–4.03 (br s, 2H), 4.02 (d, 1H), 3.94 (d, 1H), 3.46–3.34 (m, 4H), 3.30–3.26 (m, 1H), 3.24 (d, 2H), 2.89 (s, 2H), 2.84 (s, 1H).

The surrogate GPR139 agonist compound 3 had an EC_50_ of 850 nM.

#### Compound 4

The surrogate GPR139 antagonist compound 4, 1-(4-Fluoro-phenyl)-2-methyl-3-(2,2,2-trichloro-ethyl)-1,5,6,7-tetrahydro-indol-4-one, is commercially available. It was purchased from Specs (Zoetermeer, Netherlands). It had an IC_50_ of 7.4 μM, and was therefore applied at 10 μM in cell culture experiments.

None of the compounds were useful *in vivo* tools due to their unfavorable ADME properties including low whole brain exposure and brain/plasma ratio (Shi et al., 2011). Cross reactivity with the DAT or the norepinephrine transporter was determined as described (Shi et al., 2011).

##### GPR139 Calcium Mobilization Assay

**Figure 5:**
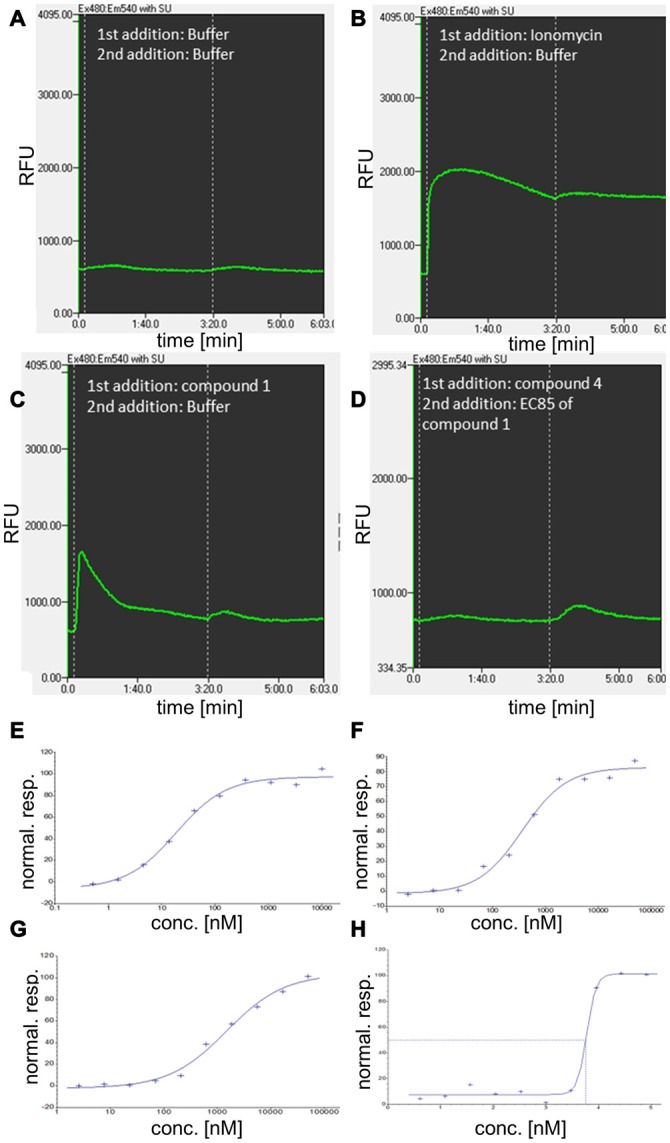
**Additional characterization of GPR139 compounds using kinetic fluorescence measurements and concentration-response determination in calcium mobilization assays. (A–D)** Examples for the kinetic of relative fluorescence units (RFU) for compound 1 and 4 and related controls, involving a two-step addition protocol. **(A)** Cellular fluorescence response to addition of buffer (approximately at time 0:00 min) and a second addition of buffer at 3:20 min. **(B)** Cellular fluorescence response to addition of the Ca^2+^ ionophore and reference compound for complete release of Ca^2+^ from intracellular stores, ionomycin. **(C)** Cellular response to addition of 10 μM compound 1 at time 0:00. **(D)** Cellular fluorescence in response to antagonist compound 4 (at 0:00, 50 μM) and addition of agonist compound 1 at EC_85_ concentration. **(E–H)** Normalized response (stimulation by agonist) for compound 1 **(E)** compound 2 **(F)**, and compound 3 **(G)** in G-protein coupled receptor 139 (GPR139) Ca^2+^ mobilization assay. **(H)** Concentration-response (inhibition by antagonist) for compound 4.

All compounds were further characterized using GPR139-based Ca^2+^ mobilization assays (Figure 5).

Standard molecular cloning techniques were used to generate Chinese hamster ovary (CHO-K1) cells stably expressing the human GPR139 receptor. The cell line was grown in Ham’s F-12K (Kaighn’s) medium (Gibco 21127), 10% FBS (Gibco 10091–155), 1% Sodium Pyruvate (Gibco 11360), 0.5 mg/ml G418 (Gibco 11811–064), 1% Penicillin Streptavidin (Gibco 15140–122). Cells expressing *GPR139* were plated in growth medium (modified to contain 5% FBS, 0.5% Penicillin/Streptavidin and 1 × ITS-X(Gibco #51500–056)) at a density of 10,000 cells/well (30 μl) in clear-bottomed, poly-D-lysine coated 384-well plates (ArcticWhite LLC, Bethlehem, PA, USA) and grown for 24 h at 37°C in the presence of 5% CO_2_. Before assaying, the cells were washed with assay buffer (Hanks’ balanced salt solution with Ca^2+^ and Mg^2+^ containing 20 mM HEPES, pH 7.4). The cells were incubated with a Ca^2+^-sensitive fluorescent dye, Calcium4 (Molecular Devices Inc., Sunnyvale, CA, USA) with 2.5 mM Probenecid (Sigma, St. Louis, MO, USA) for 50 min at 37°C and followed by 10 min at room temperature. Calcium flux was measured using a Hamamatsu FDSS7000 imaging-based plate reader (Hamamatsu Photonics) using 480 nm excitation light and emitted fluorescent light passed through a 525 nm emission filter and detected by a CCD camera. Test compounds were diluted in assay buffer from 2 or 10 mM stock solutions in 100% DMSO to give a 3× concentrated stock. Compounds were added to cells and fluorescence measured at 1 Hz starting just prior to compound addition. The fluorescence readout was calculated as max-min response, i.e., maximum fluorescence reading after and before liquid addition. The fluorescence max-min data were normalized to yield responses for no stimulation (buffer) and full stimulation (5 uM compound 1) of 0% and 100% stimulation, respectively. Antagonism was examined as inhibition of agonist-induced stimulation via a subsequent 2nd addition step of compound 1 at EC_85_ concentration (appx at time 3:20 min). The fluorescence max-min data were normalized to yield responses for no stimulation (buffer) and EC_85_ stimulation of 100% and 0% inhibition, respectively. Concentration-response data were fitted to the four-parameter logistic equation to estimate compound potency (EC_50_ or IC_50_) and efficacy (E_max_ or I_max_) (Motulsky and Christopoulos, 2003).

#### Primary DA Midbrain Neuron Culture and Toxicity Assays

**Figure 6:**
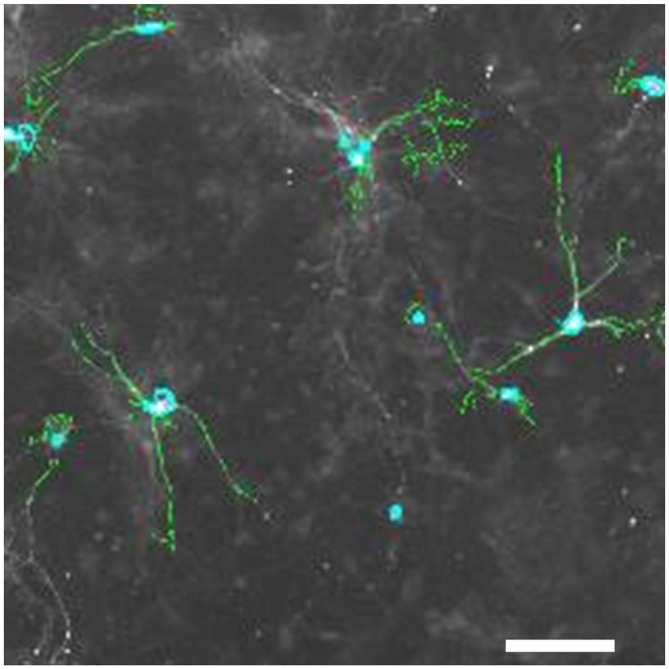
**Tyrosine-hydroxylase positive neurons in primary mesencephalic cultures.** Pixels with an intensity above a set threshold that the image analysis software detects as TH-positive are shown in green; nuclei of TH-positive cells detected by the software are marked in light blue. Space bar: 50 μM.

Experiments were conducted in accordance with the ethical guidelines of H. Lundbeck A/S and the Danish legislation of animal use for scientific procedures. Cell culture was basically performed as described (Nagel et al., 2008), with few modifications. Briefly, the mesencephalon floor was dissected from embryonic day (E) 13 mice, tissue pieces collected in HBBS, transferred to 0.1% trypsin, 0.05% DNAse (Sigma-Aldrich DN25–1G) and incubated at 37°C in a humidified atmosphere with 5% CO_2_ for 20 min; washed in DNAse, homogenized and centrifuged at 200× g, resuspended in medium (DMEM (Gibco 41965–039), 10% FCS, 1% P/S (Gibco, 15140–122), l.5% HEPES (Gibco, 15630–056), 1% Sodium Pyruvate (Gibco, 11360–039)) and plated on poly-L-lysine coated 96-well dishes. The medium was switched to neurobasal medium after 90 min (neurobasal (Gibco 21103–049) with 1% P/S, 2% B27 (Gibco, 17504–044), 1.25% 0.5 mM L-glutamine (Gibco, 25030–024)). After 6 days *in vitro* (DIV), cultures were treated with 1 μM of three different GPR139 agonists and in some experiments, concomitantly, with 10 μM of the antagonist for1 h, before exposing them to 0–1 μM MPP^+^, 0–50 μM 6-OHDA or 0–100 nM rotenone for 24 h. For the MPP^+^ treated cultures, it was also tested whether the effect of the GPR139 agonists could be blocked by concomitantly applying 10 μM GPR139 antagonist (IC_50_ 3 μM). Subsequently, cells were fixed in 4% PFA/PBS for 10 min, washed with PBS, incubated in 0.5% BSA/0.1% Triton-X/5% porcine serum for 20 min, and incubated for 24 h in rabbit anti-tyrosine hydroxylase antibodies (1:1000 in PBS, Millipore AB152); washed 2 × 5 min in PBS. Secondary antibodies (Alexa 488 rabbit) were applied 1:200 in PBS with 0.2 μg/ml Hoechst dye for 1 h and washed 2 × 5 min in PBS. One drop of DAKO fluorescent mounting medium were applied into each well. Plates were stored at 4°C until tyrosine-hydroxylase-positive (TH-positive) neurons were counted using a Thermo Scientific Cellomics^®^ ArrayScan^®^ VTI HCS Reader. An example image of such cell culture is provided in Figures 4, 6. Results were normalized against control condition and compared. Experiments were only included in the evaluation if at least 30% cell death was induced at the highest toxin concentration compared to non-treated control wells. To ensure that only healthy primary cultures were assessed, we furthermore excluded experiments with total cell counts below Mean minus standard deviation (SD) of the summarized experiments. Statistical significance of the differences between the control condition and agonist treatment was investigated by the unpaired *t* test at the 5% level using GraphPad online software.

#### Real-time Quantitative PCR

RNA was isolated using the RNAeasy kit from Qiagen, Valencia, CA, USA. Briefly, RNA was reverse transcribed and detection of PCR gene-fragments was carried out on a MJ Research light cycler by SYBR detection. The Q-PCR results were analyzed by the 2^−ΔΔCT^ method as earlier described (Pfaffl, 2001) using cyclophilin A as a control reference.

## Results

### Expression of *Gpr139* in Primary DA Midbrain Neurons

**Figure 7:**
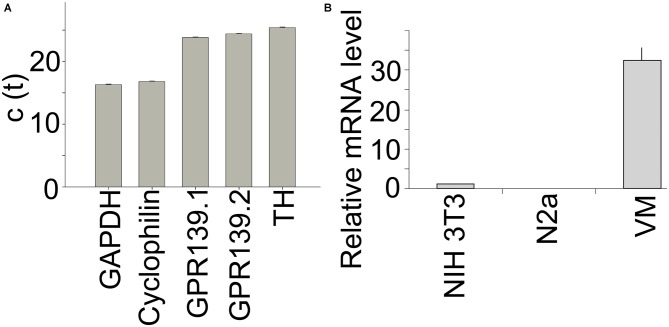
***Gpr139* is expressed in primary midbrain cultures.** Results from quantitative polymerase chain reaction (PCR) showing c(t) values for expression of **(A)** the housekeeping gene *Glyceraldehyde 3-phosphate dehydrogenase* (GAPDH); *Cyclophilin A*; *Gpr139* detected by two different primer pairs; and TH. **(B)** Expression of *Gpr139* in the mouse fibroblast cell line NIH 3T3 and the mouse neuroblastoma cell line N2a and primary midbrain cultures (VM), normalized to *Cyclophilin A*. Shown are the mean values of the experiment run in triplicates. Error bars represent standard deviations of the mean.

We used quantitative PCR to determine relative expression levels of *Gpr139* in primary midbrain neuron cultures. C(t) values using two different primer pairs for *Gpr139* were in the same range as the C(t) value for tyrosine hydroxylase, the gene characteristic for DA neurons (Figure 7A). *Gpr139* expression was about two orders of magnitude higher in primary midbrain cultures, compared to expression in a mouse fibroblast cell line, or a neuroblastoma cell line (Figure 7B).

### Protection Against Toxin-Induced Cell Death

**Figure 8:**
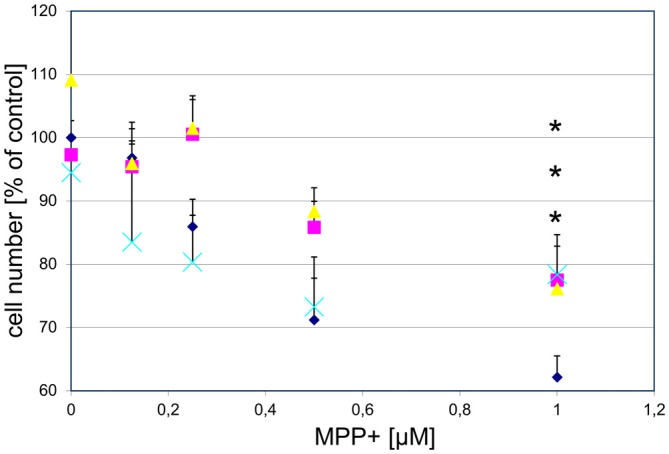
**Three different GPR139 surrogate agonists protect primary dopaminergic (DA) midbrain neurons against 1-methyl-4-phenyl-pyridinium ionMPP^+^ toxicity.** Neuronal midbrain cultures were pretreated with 1 μM of either one of the agonists compound 1 (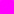), compound 2 (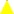), or compound 3 (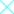), or vehicle (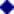) for 1 h, followed by treatment with the indicated concentrations of MPP^+^ for 24 h. TH-positive neurons were counted and normalized to numbers under control conditions. At 1 μM MPP^+^, protection by all three different agonists was significant (*p ≤ 0.05). Each data point is calculated from 12 (0 μM and 1 μM MPP^+^ concentrations); 4 (0.125 μM); 7 (0.25 μM); or 8 (0.5 μM) independent measurements.

To examine whether GPR139 agonists protect against toxin-induced DA cell death, we first treated cultured DA midbrain neurons with the GPR139 agonists and MPP^+^ . Subsequently, we determined survival of TH-positive neurons. We found that three different agonists dose-dependently and substantially protect primary DA neurons against MPP^+^ toxicity: between 40.5% (compound 2) and 42.8% (compound 3) of the cells killed by 1 μM MPP^+^ were rescued by previous incubation with 1 μM of either compound (Figure 8).

**Figure 9:**
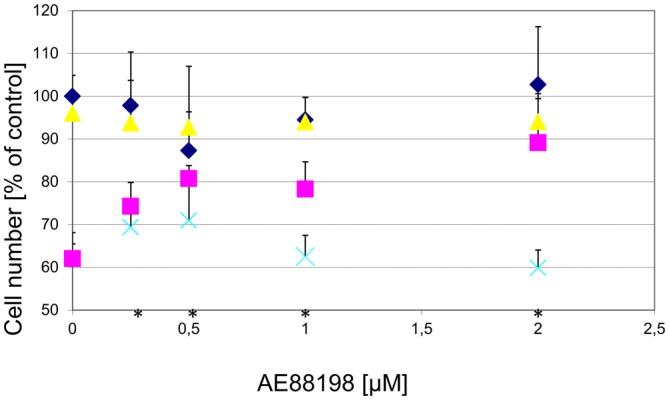
**Protection of DA midbrain neurons against MPP^+^ toxicity by a GPR139 agonist is dose dependent and can be blocked by a GPR139 antagonist.** Primary midbrain cultures were pretreated with the indicated amount of GPR139 agonist compound 3 and with either vehicle (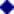), or 1 μM MPP^+^ (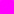); or MPP^+^ with 10 μM of the antagonist compound 4 (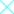), or with the antagonist compound 4 alone (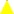). The number of TH-positive neurons was determined 24 h later and normalized to control conditions. Statistical significance compared to vehicle treated cells is indicated (*p < 0.05).

To determine whether the observed protection acted specifically on GPR139, we tested whether the effect was reversible by a GPR139 antagonist. While the antagonist alone did not have an effect on DA cell survival, the antagonist blocked the protective effect of the agonists (Figure 9).

**Figure 10:**
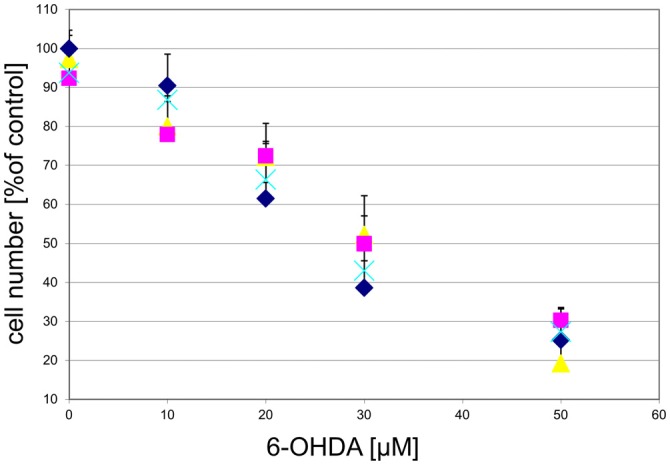
**GPR139 agonists do not protect cultured DA midbrain neurons against 6-OHDA toxicity**. Neuronal midbrain cultures were pretreated with 1 μM of either one of the agonists compound 1 (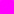), compound 2 (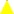), or compound 3 (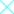), or vehicle (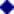) for 1 h, followed by treatment with the indicated concentrations of 6-hydroxydopamine (6-OHDA) for 24 h. TH-positive neurons were counted and normalized to control numbers.

To determine whether the GPR139 agonists would also protect against other toxins affecting DA neurons, we examined the effect of the agonists on 6-OHDA toxicity. We found that GPR139 agonists did not protect cultured DA midbrain neurons against extended culture periods or 6-OHDA (Figure 10).

**Figure 11:**
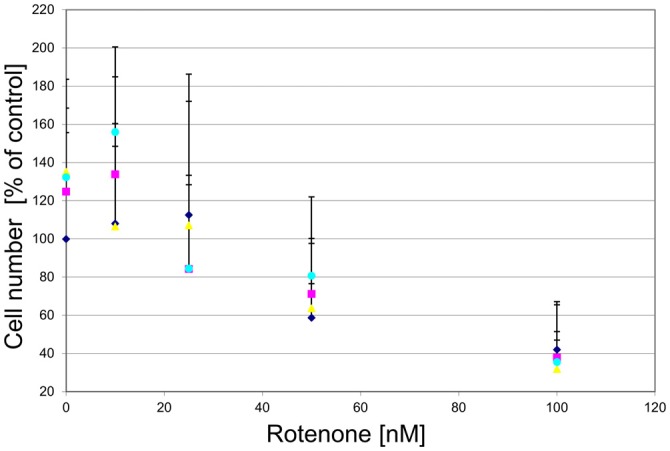
**GPR139 agonists do not protect cultured DA midbrain neurons against rotenone toxicity.** Mesencephalic cultures were treated with 1 μM of either one of the GPR139 agonists compound 1 (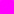), compound 2 (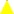), or compound 3 (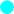), or vehicle (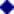) for 1 h before exposing them to 0–100 nM of rotenone. Results are average of ± SEM (*n* = 4 independent experiments). TH-positive neurons were counted and normalized to control numbers (100%). At a given rotenone concentration, none of the values among the treatment groups were significantly different from one another.

To examine whether the agonist-induced protection likely was mediated via mitochondrial complex I inhibition, we treated the cultured DA midbrain neurons with rotenone, which, like MPP^+^, is a mitochondrial complex I inhibitor. While rotenone did induce a dose-dependent cell death in the TH-positive neurons, no rescue was seen in the agonist treated sub-population (Figure 11).

### Agonist-Dopamine Transporter (DAT) Interaction

As both 6-OHDA and MPP^+^ are taken up via the DAT, it was not likely that the agonists mediated their protection against MPP^+^ by merely blocking the DAT. However, to further exclude the possibility that the protection against MPP^+^ by the agonist compound 3 is mediated by blocking MPP^+^ uptake, rather than signaling through GPR139, DA and noradrenaline uptake in the presence of the agonist was tested. At 10 μM compound 3 inhibited DA uptake only 13% and norepinephrine uptake only −13%, suggesting that the protective effect observed with the agonist is likely not mediated by directly acting on the DAT. Ten micrometres (10 μM) compound 1 inhibited binding to the DAT or the norepinephrine transporter by 4% or −8%, respectively (Shi et al., 2011) compound 2 inhibited binding to the DAT or the norepinephrine transporter 2% or −4%, respectively.

We next sought to examine whether the agonists would also provide neuroprotection *in vivo*. However, we found that ADME properties of the compounds were not favorable to provide sufficient brain exposure and receptor occupancy, neither by oral application, nor by subcutaneous delivery via osmotic pumps (data not shown).

To confirm the motor deficits earlier described in *Gpr139* KO mice, the mice underwent behavioral testing on a rotarod with a fixed and increasing speed and a balance beam paradigm. We could not detect any differences among *Gpr139* KO mice, heterozygous mice, or wt mice (data not shown). This may, however, be due to the fact that the mice were on a non-congenic background different from the ones used in earlier studies (Murphy and Croll-Kalish, 2004).

## Discussion

We demonstrate that three GPR139 agonists dose-dependently protect primary DA neurons against MPP^+^ toxicity. When treating cultured DA midbrain neurons with rotenone or 6-OHDA, we also observed dose-dependent cell death; however, GPR139 agonist treatment did not rescue the neurons. Moreover, no protection was demonstrated against prolonged culture periods, suggesting that GPR139 agonism does not enhance general cellular viability and resistance against apoptotic stimuli.

Previously, not peer-reviewed work suggested that GPR139 KO mice display a deficit in motor performance (Murphy and Croll-Kalish, 2004). However, in pilot experiments, we could not confirm those deficits using the rotarod and the balance beam test. That discrepancy might be due to a different and variable background of the mice examined compared to the mice in the earlier study.

Work by Song et al. (2012) has previously reported that the bibenzyl compound Chrysotoxine could antagonize the toxicity of MPP^+^, but not rotenone in SH-SY5Y cells. However, the authors explain that the MPP^+^ protection is, at least partly, due to inhibition of the DAT (Song et al., 2012). In our study the protective effect of the agonists seen in the MPP^+^ treated cultures is unlikely to be due to DAT inhibition. First, 6-OHDA is also partly taken up via the DAT and no protection of the agonists was seen when cultures were treated with 6-OHDA. Next, agonist compound 1 and compound 2 did not show cross-reactivity with the DAT. It cannot be entirely ruled out that compound 3 does interfere with the DAT; however, considering the very similar effect of the three agonist on MPP^+^ toxicity, that mechanism is not very likely.

We further examined the specificity of the agonist protection against MPP^+^ toxicity by showing that a GPR139 antagonist could block the protection, while the antagonist itself was not toxic to DA neuron cultures. Thus, together with the fact that three different agonists showed similar effects, and the low cross-reactivity of the compounds in a broad pharmacology panel screen, assaying the ability to displace radioligand binding to the assayed targets (Shi et al., 2011), it is likely that the protection we observed is mediated through GPR139.

Our data complement previous findings on the mechanism of GPR139 signal transduction. Most studies, including recent ones from our own group (Matsuo et al., 2005; Süsens et al., 2006; Shi et al., 2011; Isberg et al., 2014; Dvorak et al., 2015; Liu et al., 2015) suggest that the receptor mobilizes intracellular calcium, which can be blocked with the G_q_ inhibitors YM-254890 (Matsuo et al., 2005) and UBO-QIC (Isberg et al., 2014), which is characteristic of G_q_ pathway activation. Moreover, as shown in Figure 5, the GPR139 agonists used in this study also activate Ca^2+^ mobilization. Downstream of Ca^2+^ activation, there are a “myriad” (Dunn and Ferguson, 2015) of mechanisms that control GPCR signaling and trafficking. Which of those signal transduction pathways are relevant upon GPR139 activation remains to be investigated. There are also reports on additional activation of the G_i_ (Süsens et al., 2006) or the G_s_ (Hu et al., 2009) pathways. It can thus not be ruled out that GPR139 might also activate other pathways under certain circumstances, further indicating the complexity of those downstream events.

A few previous studies have shown differences in the effects of MPP^+^ and rotenone. While both are mitochondrial complex I inhibitors, rotenone is more potent (Higgins and Greenamyre, 1996; Mizuno et al., 1987). Rotenone is lipophilic, and it can thus readily cross the cell membrane, whereas MPP^+^ depends on the DAT for transport into cells (Javitch et al., 1985). One could speculate that due to its easy entry to cells rotenone would likely induce cell death in the entire cell population it becomes exposed to. Indeed, both *in vivo* and *in* *vitro* rotenone has been found to result in unspecific DA and non-DA neuronal cell death in some studies (Nakamura et al., 2000; Tieu, 2011). However, using different experimental approaches, DA neurons are particularly vulnerable (Ferrante et al., 1997; Betarbet et al., 2000; Bywood and Johnson, 2003; Kweon et al., 2004; Radad et al., 2008). It has been suggested that chronic exposure to low MPP^+^ (Nakamura et al., 2000) or rotenone (Kweon et al., 2004) concentrations is selectively more toxic to DA neurons, whereas acutely administered high concentrations cause less discriminative cell death. DA neurons are more vulnerable to mitochondrial energy disruption than non-DA neurons (Kweon et al., 2004), which might explain why DA neurons can be selectively killed by rotenone.

While we observed an average DA neuronal cell death in the primary cultures of 60% after 24 h with 100 nM rotenone and 40% with 1 μM MPP^+^, in our experience it also takes nM MPP^+^ concentrations to achieve 50% cell death in the neuroblastoma SH-SY5Y cell line (not shown). Nevertheless, as those cells express DA markers, they also provide a useful model system to study the mechanism of toxicity induced by substances used in PD models. Giordano et al. (2012) found a number of differences in the effects of MPP^+^ and rotenone on bioenergetics and cell death in differentiated SH-SY5Y neurons. 50% cell death was obtained after 24 h incubation with 5 nM rotenone, 5 mM MPP^+^ or 100 μM 6-OHDA. Increasing doses of rotenone resulted in significant cell death and caspase 3 activation. Rotenone immediately inhibited the mitochondrial basal oxygen consumption rate (OCR) with a resulting decrease of ATP-linked OCR, reserve capacity and a stimulation of glycolysis. With high doses of MPP^+^ nearly eliminating basal and ATP-linked OCR, less pronounced cell death was seen compared to that induced by rotenone. Cytotoxic 6-OHDA doses had much lower impact on bioenergetics functions and thus Giordano et al. (2012) suggests that its toxic effect is probably independent of these (Giordano et al., 2012).

While the inhibition of the mitochondrial complex I plays a substantial role in both the toxicity of MPP^+^ and rotenone (Sherer et al., 2003; Richardson et al., 2007), several studies have suggested that it cannot account for the entire toxic effect seen in DA neurons (Nakamura et al., 2000; Kweon et al., 2004; Choi et al., 2008). It is thus possible that GPR139 agonists have different effects in the two model systems due to the differential effects of MPP^+^ and rotenone on mitochondrial complex I inhibition, and on the other hand by acting on pathways independent of those. Whether GPR139 activation e.g., inhibits the apoptotic pathway, for instance by interfering with molecules of the Bcl-2 family, or by stabilizing mitochondrial integrity, etc., should be addressed by future experiments.

Although neuroprotection against MPP^+^, rotenone, or 6-OHDA often does not translate into the identification of PD treatment targets or drug candidates, it does go beyond merely providing a model system to study the effects of specific elimination of DA neurons. Mitochondrial dysfunction is likely to also play an important role in PD (Schapira and Gegg, 2011). Moreover, exposure to rotenone and other pesticides in farming communities substantially increases the incidence of PD (Tanner et al., 2011; Kamel et al., 2014). Thus, understanding the mechanisms of toxin-induced DAergic neuronal death could also contribute to a deeper understanding of some aspects of disease mechanisms.

In conclusion, the difference in protection against MPP^+^ and rotenone might be explained by a more pronounced bioenergetic effect of rotenone toxicity, and other pathways affected by the toxins beyond mitochondrial complex I inhibition. The protective effect of 3 different agonists against MPP^+^ could be reversed by a GPR139 antagonist. Together with the missing protection against 6-OHDA this points towards a specific protective effect of the agonists mediated through GPR139. Our results further substantiate differences in the effect of three of the most commonly used toxins in PD models.

As described earlier by Shi et al. (2011) agonist compound 1 presented in this study does not hold the ADME properties necessary for *in vivo* testing. The same applies to agonists compound 2 and compound 3. While this manuscript was in preparation, a selective, high-affinity GPR139 small molecule agonist with favorable ADME properties and high brain exposure was developed (Dvorak et al., 2015). It was found that that agonist reduced rat motor activity *in vivo* (Liu et al., 2015). To validate whether activation of GPR139 *in vivo* has a protective effect on DA neurons that GPR139 agonist could be used in MPTP studies in mice to evaluate rescue of DA neurons in agonist treated animals. Furthermore, detailed characterization of the GPR139 KO mouse on a congenic background may render additional information on GPR139 function.

## Author Contributions

All authors finally approved the version to be published and agreed to be accountable for all aspects of the work. KBA acquired and analyzed the data on rotenone neuroprotection as well as the agonist *in vivo* data, and wrote the initial draft of the manuscript. JLJ acquired and analyzed the data on *Gpr139* expression, analyzed that data, and wrote the corresponding section of the work. MH acquired and analyzed the data on compound screening, cross-reactivity and EC_50_ determination, and wrote the corresponding sections of the manuscript. GPS did the conception to generate, analyze and identify the compounds, and wrote the corresponding section of the manuscript. GPHD initiated and designed the concept of the work; was involved in the data generation on MPP^+^ and 6-OHDA toxicity and revised and wrote the final versions of the manuscript.

## Conflict of Interest Statement

While this work was in progress, all authors were employed and paid by the pharmaceutical company H. Lundbeck A/S in Valby, Denmark, which focuses on research, development, production, marketing and sale of medication for the treatment of psychiatric and neurological diseases.

## Abbreviations

ADME: absorption, distribution, metabolism, and excretion
6-OHDA: 6-hydroxydopamine
BBB: blood-brain barrier
DMEM: Dulbecco’s Modified Eagle Medium
DA: Dopaminergic
DAT: Dopamine transporter
DIV: Days *in vitro*
GPCR: G protein-coupled receptor
GPR139: G protein-coupled receptor 139
HBSS: Hank’s Balanced Salt Solution
MPP^+^: 1-methyl-4-phenyl-pyridinium ion
MPTP: 1-methyl-4-phenyl-1 2,3,6-tetrahydropyridine
OCR: Oxygen consumption rate
PD: Parkinson’s disease
P/S: Penicillin/Streptomycin Solution
SN: Substantia nigra
SNpc: Substantia nigra pars compacta
TH: Tyrosine hydroxylase
TMS: tetramethylsilane.
